# Expression of B4GALNT1, an essential glycosyltransferase for the synthesis of complex gangliosides, suppresses BACE1 degradation and modulates APP processing

**DOI:** 10.1038/srep34505

**Published:** 2016-09-30

**Authors:** Tokiaki Yamaguchi, Yoshio Yamauchi, Keiko Furukawa, Yuhsuke Ohmi, Yuki Ohkawa, Qing Zhang, Tetsuya Okajima, Koichi Furukawa

**Affiliations:** 1 Department of Biochemistry II, Nagoya University Graduate School of Medicine, 65 Tsurumai, Showa-ku, Nagoya 466-8550, Japan; 2 Department of Biomedical Sciences, Chubu University College of Life and Health Science, Kasugai 487-8501, Japan

## Abstract

Alzheimer’s disease (AD) is the most prevalent form of dementia characterized by the extracellular accumulation of amyloid β (Aβ) peptides, which are produced by proteolytic cleavages of amyloid precursor protein (APP). Gangliosides are involved in AD pathophysiology including Aβ deposition and APP processing, yet the detailed mechanisms are not fully understood. Here we examined how changes in the carbohydrate moiety of gangliosides alter APP processing in human melanoma cells, neuroectoderm-derived cells. We showed that forced expression of GD2, GM2 or GM1 (by introducing B4GALNT1 cDNA into cells not expressing this glycosyltransferase) results in increases of α- and β-site cleavages of APP with a prominent increase in β-cleavage. We also showed that β-site APP cleaving enzyme 1 (BACE1) protein is highly protected from the degradation in cells expressing these gangliosides, thereby increasing the expression of this protein. Unexpectedly, adding gangliosides exogenously altered neither BACE1 levels nor β-site cleavage. The stabilisation of BACE1 protein led to the increase of this protein in lipid rafts, where BACE1 processes APP. Based on the current results, we propose a hitherto undisclosed link between ganglioside expression and AD; the expression of B4GALNT1 positively regulates the β-site cleavage by mainly inhibiting the lysosomal degradation of BACE1 protein.

Alzheimer’s disease (AD) is the most common form of dementia characterized by memory loss and cognitive decline. The extracellular accumulation of amyloid plaques and the intracellular accumulation of neurofibrillary tangles are pathological hallmarks of this disease^1^,^2^. The plaques are composed of aggregated amyloid-β (Aβ) peptides, which are generated by two proteolytic cleavages of amyloid precursor protein (APP) at the β- and the γ-sites. The generation of Aβ requires the two membrane-bound proteases β-secretase and γ-secretase. γ-secretase is a multi-protein complex; presenillin 1 (PSEN1) is the catalytic component of this complex^3^. β-site APP cleaving enzyme 1 (BACE1) serves as the major enzyme for the β-site cleavage^4^. To generate Aβ, APP is first cleaved by BACE1 in the acidic compartments, and the resultant β-C-terminal fragments (CTFs) are then cleaved by γ-secretase. The increase in Aβ production is an early event of AD pathogenesis. It has been reported that Aβ accumulation proceeds 15 years before the symptom appears^5^. Therefore, suppression of Aβ generation and deposition is expected as one of the most promising strategy to prevent AD^6^.

Gangliosides are glycosphingolipids containing one or more sialic acids, and play various important roles in the body. The brain contains a wide variety of gangliosides, and complex gangliosides play crucial roles in the maintenance of integrity of the nervous system and in neurodevelopment^7^,^8^,^9^. The two glycosyltransferases ST8SIA1 (also known as GD3 synthase or α-2,8 sialyltransferase) and B4GALNT1 (also known as GM2/GD2 synthase or β-1,4 *N*-acetylgalactosaminyltransferase 1) are required for the biosynthesis of complex gangliosides^10^; ST8SIA1 transfers a sialic acid to GM3, yielding GD3, while B4GALNT1 transfers a *N*-acetylgalactosamine (GalNAc) to GM3 or GD3, yielding GM2 or GD2, respectively. These two enzymes enable cells to synthesize a-series and b-series complex gangliosides. Evidence shows that gangliosides are involved in pathophysiology of AD^11^,^12^. GM1, one of the most abundant gangliosides in the brain, binds to Aβ at the cell surface, which accelerates extracellular deposition of the peptides^13^,^14^. In addition to GM1, various gangliosides have an ability to interact with Aβ^15^. In AD model mice, depletion of the *St8sia1* gene reduced Aβ deposition in the brain and improved memory^16^. In contrast, it was reported that GD3 enhances Aβ production when added exogenously^17^. Importantly, changes in composition of brain gangliosides were observed in patients with AD^18^,^19^,^20^,^21^, implicating that gangliosides directly associate with pathophysiology of AD. However, how the changes in ganglioside composition occur in the patients and how the changes are involved in the disease remain poorly understood.

Most studies performed to date examined effects of gangliosides on AD-associated events including APP processing and Aβ deposition by adding one or more gangliosides exogenously or by inhibiting the first step in glycosphingolipid synthesis in cell culture systems^17^,^22^,^23^,^24^. Exogenously added gangliosides may behave differently from those endogenously synthesized. Incorporated gangliosides might be processed in cells. Also, another possibility that a glycosyltransferase(s) involved in ganglioside synthesis itself affects an AD-associated event cannot be excluded. In the present study, cells synthesizing different gangliosides are established by forcedly expressing one or two glycosyltransferases, and we then make use of these glycan-remodeled cells to examine whether endogenously synthesized gangliosides modulate APP processing. Our results show that B4GALNT1 expression prolongs the half-life of BACE1 protein and enhances the β-cleavage of APP. Our findings thus reveal a previously undisclosed link between ganglioside metabolism and AD.

## Results

### Effects of endogenously synthesized gangliosides on APP processing

**Figure 1:**
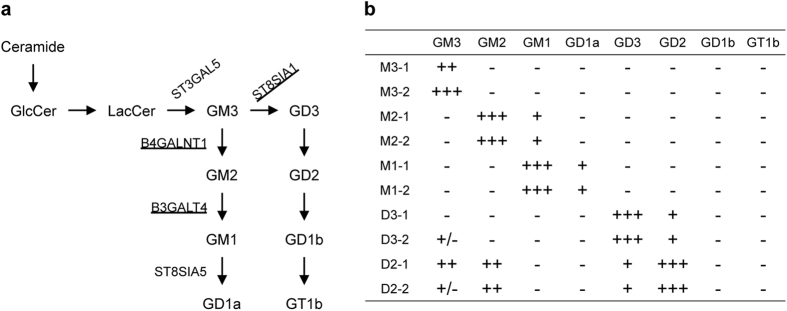
Expression of gangliosides in cells used in this study. (**a**) Biosynthetic pathways of gangliosides and glycosyltransferases involved in their synthesis. Glycosyltransferases forcedly expressed in this study are underlined. (**b**) Ganglioside expression. Cell surface expression of gangliosides in the transfectants was examined by FACS as described in Methods. Levels of ganglioside expression are summarized based on the result of Supplemental Figure 1.

Previous works showed that in cell culture systems, exogenous gangliosides modulate APP processing^17^,^22^,^23^,^24^. To further characterize a role of gangliosides in APP processing, we established cells that endogenously synthesize and express GM3, GM2, GM1, GD3 or GD2 as the major gangliosides, by forcedly expressing one or two glycosyltransferases (Fig. 1a). As recipient cells, we used a human melanoma cell line, which is a neuro-ectoderm origin. The expression profiles and compositions of gangliosides in the transfectants are shown in Supplemental Figure 1a,b, and are summarized in Fig. 1b. Mock transfectants (designated as M3-1 and M3-2) expressed GM3. Introduction of ST8SIA1 cDNA resulted in the high expression of GD3 (designated as D3-1 and D3-2)^25^,^26^. Forced expression of B4GALNT1 in GM3-expressing cells or GD3-expressing cells led to the expression of GM2 (designated as M2-1 and M2-2 cells) or GD2 (designated as D2-1 and D2-2 cells) at high levels, respectively. Forced B3GALT4 (GM1/GD1b synthase) expression in GM2-expressing cells resulted in the expression of GM1 and GD1a (designated as M1-1 and M1-2 cells).

**Figure 2:**
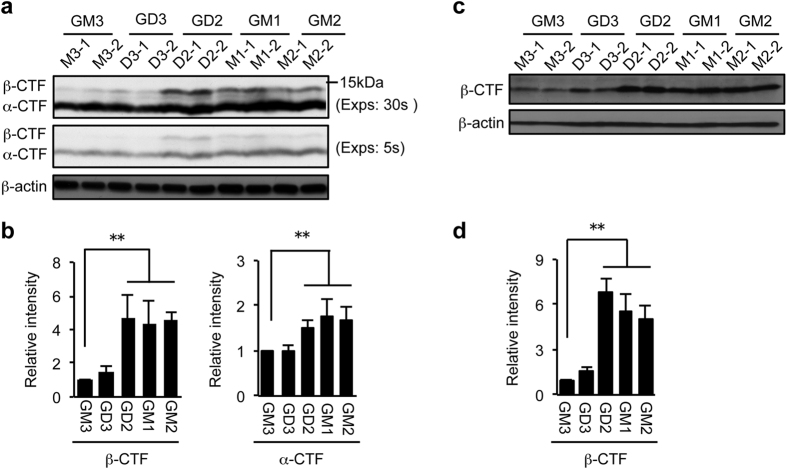
Effect of ganglioside expression on APP processing. Whole cell lysate was prepared from cells treated with 1 μM DAPT for 12 h. Equal amounts of cellular proteins were subjected to immunoblot analysis with anti-APP antibody Y188 (**a**) or 82E1(**c**). Longer (30 sec) and shorter (5 sec) exposure images are shown in panel a. β-actin served as an internal standard. Levels of α-CTF and/or β-CTF shown in panels a and c were quantified and presented in panels b and d, respectively. Data represent means ± s.d. (n = 6). Statistical analysis was performed by one-way ANOVA with Tukey-Kramer post hoc test (**P < 0.01).

APP is first cleaved at the α-site or the β-site, producing α-CTF or β-CTF, respectively. These two CTFs then become the substrates of γ-secretase. To validate the effect of endogenously synthesized gangliosides on APP processing, we first examined levels of α- and β-CTFs in cells expressing different gangliosides described above. Because α-CTF and β-CTF are rapidly cleaved by γ-secretase, we treated cells with the γ-secretase inhibitor DAPT to evaluate the α- and β-site cleavages. DAPT treatment resulted in approximately 10-fold increases in both CTFs (Supplemental Fig. 2). In GD2-, GM2- and GM1-expressing cells, β-CTF was increased more than 4-fold compared to GM3-expressing cells (Fig. 2a,b). In contrast, level of β-CTF was unchanged in GD3-expressing cells. The α-cleavage of APP was also significantly increased in cells expressing GD2, GM2 or GM1, but the increase was modest compared to the β-cleavage. To further confirm the result, we used the monoclonal antibody 82E1, which specifically recognizes β-CTF, but not α-CTF. The result clearly showed that the level of β-CTF is markedly increased in cells expressing GD2, GM2 or GM1 (Fig. 2c,d). Although we attempted to examine the products of γ-site cleavage in these cells by using the ELISA systems, both Aβ40 and Aβ42 were under detection limit (data not shown). The results in Fig. 2 suggest that the expression of GD2, GM2, or GM1 enhances the β-site cleavage of APP.

### Effects of gangliosides on the expression of APP and BACE1

**Figure 3:**
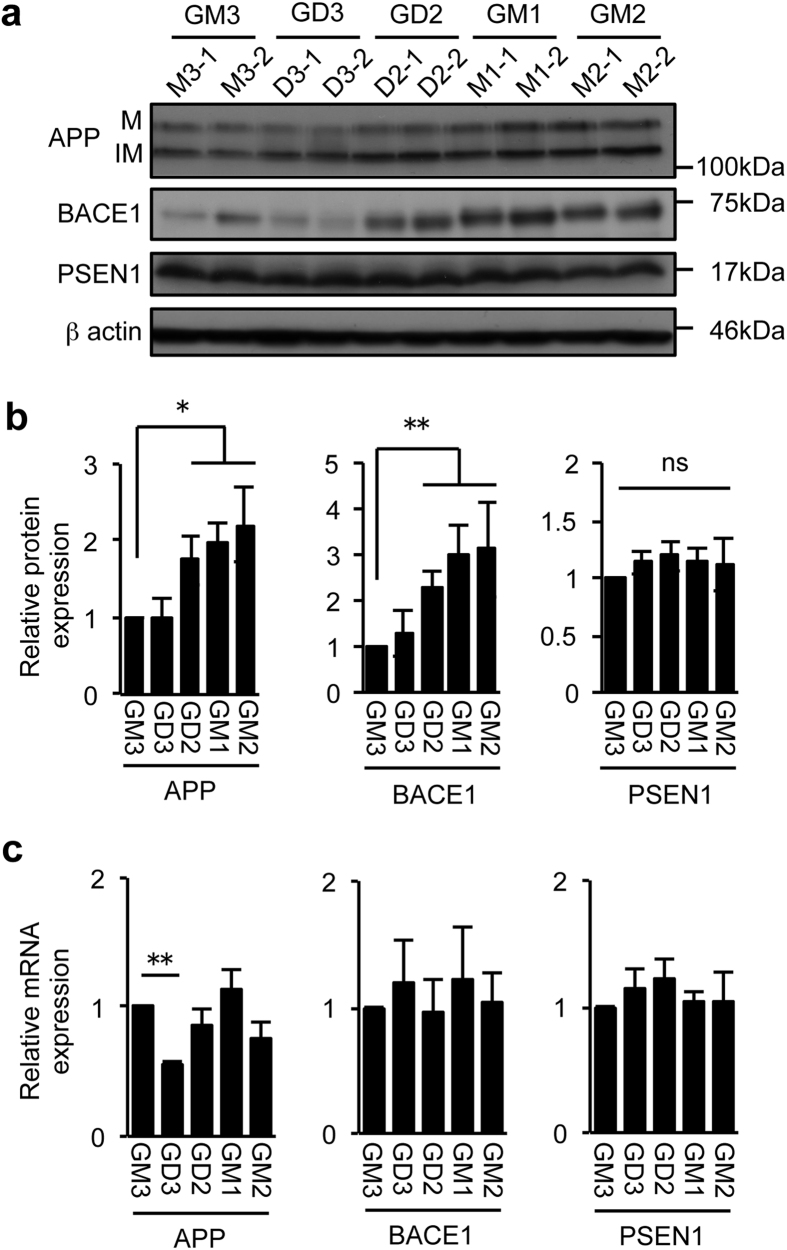
Expression of APP, BACE1 and PSEN1. (**a**,**b**) Protein expression levels. Cell lysate was subjected to immunoblot analysis with antibodies to APP (22C11), BACE1, or PSEN1. The expression levels were quantified and plotted in panel b. Data shown are means ± s.d. (n = 6 for APP and PSEN1, n = 8 for BACE1). Statistical analysis was performed by one-way ANOVA with Tukey-Kramer (APP and BACE1) or Dunnett (PSEN1) post hoc test. *P < 0.05, **P < 0.01. IM, immature form; M, mature form (**c**) mRNA expression. Levels of APP, BACE1, and PSEN1 mRNAs were analysed by quantitative RT-PCR as described in Methods. Data shown are means ± s.d. (n = 3 for APP and PSEN1, n = 5 for BACE1). Statistical analysis was performed by one-way ANOVA with Tukey-Kramer (APP) or Dunnett (BACE1 and PSEN1) post hoc test. *P < 0.05, **P < 0.01. ns; not significant.

The increase in β-CTF in the presence of DAPT in cells expressing the specific gangliosides could be explained by the following two possibilities; an increase in APP expression and/or in BACE1 expression/activity. To clarify this issue, we examined the expression of APP, BACE1 and the processed, active-form of PSEN1. The results showed that in cells expressing GD2, GM2 or GM1, BACE1 protein expression was increased more than 2 to 3-fold compared to GM3-expressing cells (Fig. 3a,b). We also found an increase in APP protein in these cells. On the other hand, gangliosides unaffected PSEN1 expression. To determine whether the increase in BACE1 protein is due to an increase of the transcription, we compared mRNA levels of BACE1 as well as those of APP and PSEN1. The results showed that BACE1 and PSEN1 mRNA levels were at similar levels among the cell lines (Fig. 3c). APP mRNA expression was not increased in GD2-, GM2-, or GM1-expressing cells compared to GM3-expressing cells, but it was significantly reduced in GD3-expressing cells. Together, these results support the hypothesis that the increased protein expression of BACE1 is involved in the enhancement of the β-site cleavage of APP in cells expressing GD2, GM2 or GM1. In addition, the increase of α- and β-CTF may also be caused by the increase in the APP protein expression.

**Figure 4:**
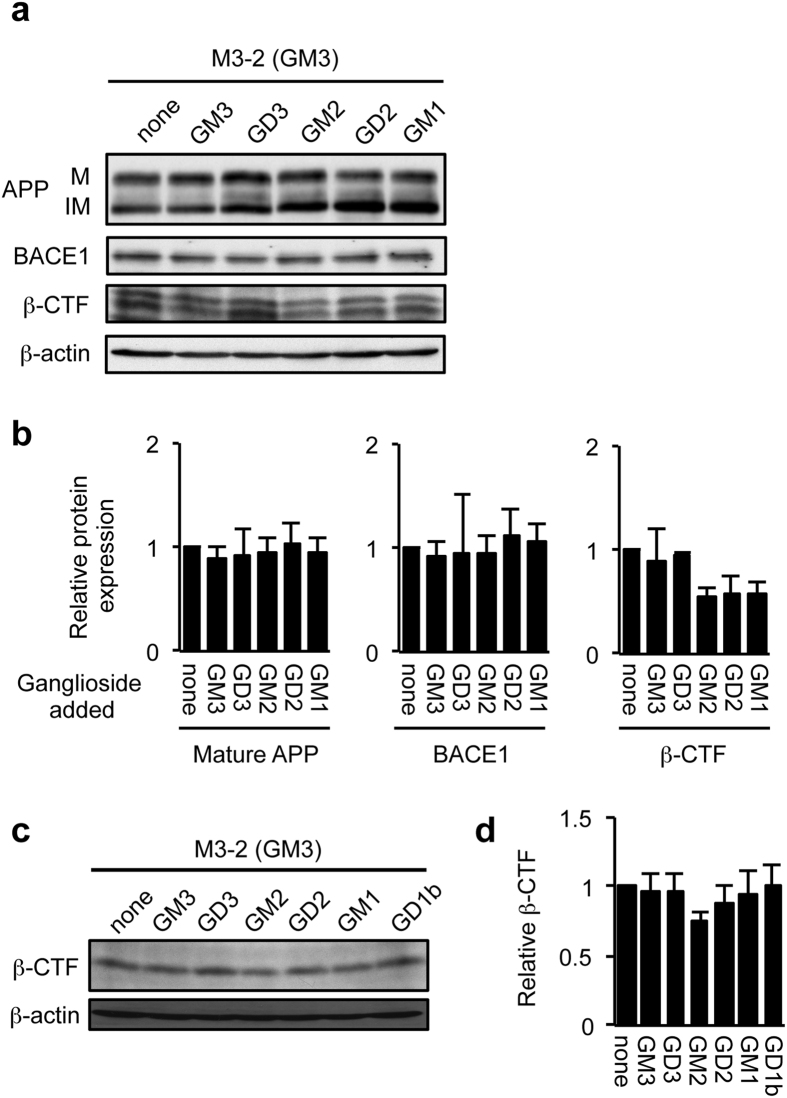
Effect of exogenous gangliosides on BACE1 expression and activity. (**a,b**) BACE1 protein expression. GM3-expressing cells (M3-2) were incubated without or with GM3, GD3, GM2, GD2 or GM1 (30 μM each) for 21 h. Whole cell lysate was subjected to immunoblot analysis for BACE1, APP (22C11) and β-CTFs (Y188). β-actin served as an internal standard. BACE1, APP and β-CTF levels were quantified and plotted in panel b. Data are means ± s.d. (n = 3). Statistical analysis was performed by one-way ANOVA with Dunnett post hoc test, and statistical significance was not detected. IM, immature form; M, mature form. (**c,d**) Generation of β-CTF. GM3-expressing cells (M3-2) were treated with gangliosides as above. Twelve hours before harvesting cells, DAPT was added to culture medium at the final concentration of 1 μM. Whole cell lysate was subjected to immunoblot analysis with anti-Aβ (82E1) that detects only β-CTF. β-CTF level was quantified and plotted in panel d. Data represent means ± s.d. (n = 3). Statistical analysis was performed by one-way ANOVA with Dunnett post hoc test, and statistical significance was not detected.

GD2, GM2 and GM1 all contain a β1, 4-linked GalNAc residue on the galactose, while this structure is not present in GM3 and GD3. The results described above suggested that gangliosides containing a GalNAc residue modulate BACE1 protein expression. Since the majority of gangliosides reside in the plasma membrane, we asked whether cell surface gangliosides have an ability to alter BACE1 expression. To test this hypothesis, we loaded five different gangliosides, GM3, GD3, GM2, GD2 or GM1 exogenously onto the cell surface of GM3-expressing cells, and assessed BACE1 protein expression. Unexpectedly, despite the successful incorporation (Supplemental Fig. 3) and cell surface expression (data not shown) of exogenous gangliosides, none of these gangliosides increased BACE1 and APP protein expression (Fig. 4a,b). Consistent with this result, all these gangliosides did not affect β-CTF levels (Fig. 4c,d). These results suggest that expression of B4GALNT1 protein plays an important role in the regulation of BACE1 expression and activity, while the possibility that GalNAc-containing gangliosides are involved in these processes cannot be excluded.

### Stability of BACE1 protein

**Figure 5:**
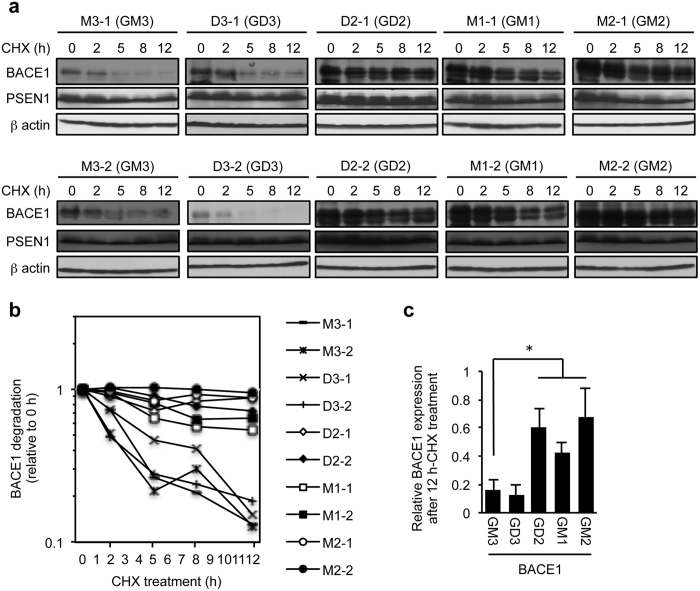
Stabilisation of BACE1 in cells expressing B4GALNT1. (**a**) Cells set up as in Fig. 2 were treated with 150 μM cycloheximide (CHX) for 2, 5, 8 or 12 h. Whole cell lysate was subjected to immunoblot to examine BACE1 and PSEN1 protein levels. (**b**) Relative changes in BACE1 protein levels in the cells treated with CHX were plotted based on panel (a). (**c**) Relative BACE1 protein levels in cells expressing the indicated gangliosides after the CHX treatment for 12 h. Data represent means ± s.d. (n = 8). *P < 0.05 by one-way ANOVA with Tukey-Kramer post hoc test.

The results described in Figs 3 and 4 suggest that B4GALNT1 expression post-transcriptionally modulates BACE1 protein expression. It was reported that BACE1 degradation is highly regulated^27^. We thus examined the stability of BACE1 protein in the presence or absence of B4GALNT1 expression. Cells expressing or not expressing B4GALNT1 were treated with cycloheximide, an inhibitor of protein synthesis, for various time, and BACE1 and PSEN1 protein levels were assessed by immunoblotting (Fig. 5a). The results showed that BACE1 protein is rapidly degraded with a half-life of less than 5 h in cells not expressing B4GALNT1 (GM3- and GD3-expressing cells) (Fig. 5b). In contrast, BACE1 protein was highly protected from degradation in cells expressing B4GALNT1 (GD2-, GM2-, GM1-expressing cells); the half-life was prolonged to more than 12 h in the cells expressing B4GALNT1 (Fig. 5b,c). On the other hand, it was shown that PSEN1-CTF is a long-live protein as previously reported^28^. Neither B4GALNT1 nor gangliosides affected the stability of PSEN1.

**Figure 6:**
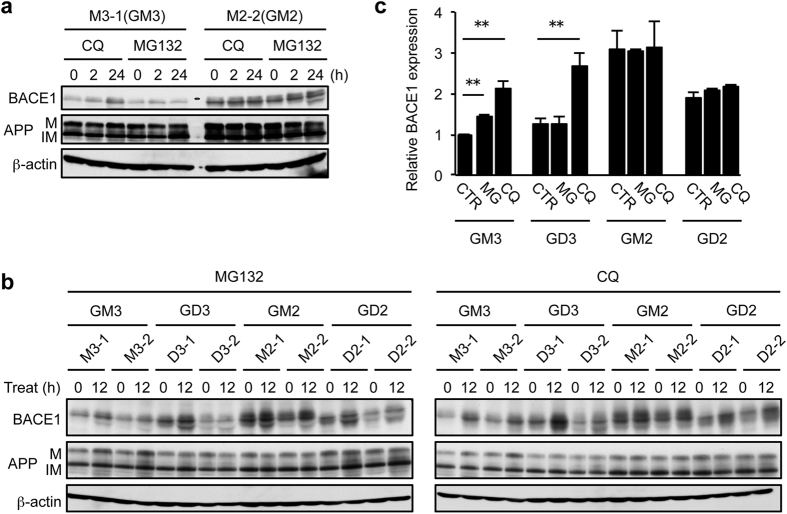
Effect of lysosome and proteasome inhibitors on BACE1 expression. (**a**) Cells expressing GM3 (M3-1) or GM2 (M2-2) were treated with 10 μM chloroquine (CQ) or 10 μM MG-132 for the indicated time. Whole cell lysate was subjected to immunoblot to examine BACE1 and APP expression. β-actin served as an internal standard. IM, immature form; M, mature form. (**b,c**) Cells were treated with either 10 μM MG-132 (MG) or 10 μM chloroquine (CQ) for 12 h. BACE1 and APP expression was examined as above (**b**). BACE1 expression relative to GM3-expressing cells was plotted in panel c. Data represent means ± s.d. (n = 6). **P < 0.01 by Student’s t-test. CTR, control.

BACE1 is degraded by the lysosomal and proteasomal pathways^29^,^30^. To determine which pathway is affected by B4GALNT1 expression, we treated GM3- and GM2-expressing cells with the lysosomal inhibitor chloroquine or the proteasome inhibitor MG-132 for 2 or 24 h, and examined BACE1 protein expression. The results showed that chloroquine, but not MG-132, markedly increases BACE1 protein in GM3-expressing cells (Fig. 6a), identifying the lysosomal pathway as a major BACE1 degradation mechanism in these cells. In contrast to GM3-expressing cells, chloroquine had no effect on BACE1 expression in cells expressing GM2. To further confirm this result, we next compared the effect of these two inhibitors on BACE1 expression among cells expressing GM3, GD3, GM2 or GD2. The results showed that chloroquine, but not MG-132, increases BACE1 expression only in GM3- and GD3-expressing cells (Fig. 6b,c). In cells expressing B4GALNT1, BACE1 expression was unchanged not only by MG-132 but also by chloroquine. These results suggest that B4GALNT1 expression protects BACE1 from its lysosomal degradation.

### Localisation of BACE1 in lipid rafts

**Figure 7:**
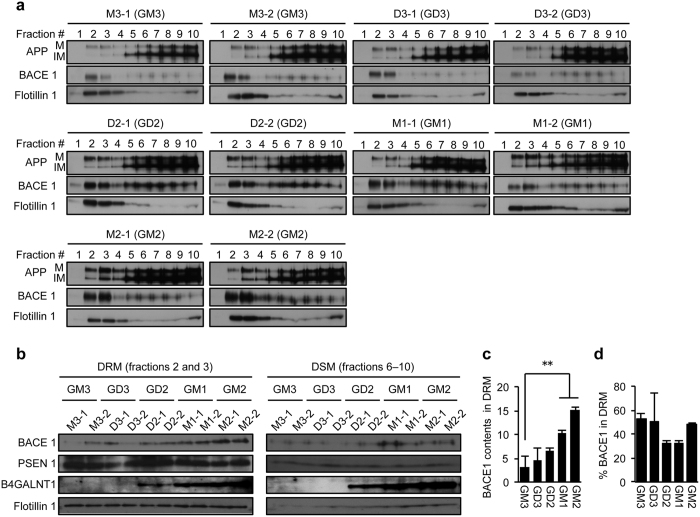
Abundance of BACE1 protein in detergent-resistant membrane domains. (**a**) Distribution of APP and BACE1 in DRM and DSM. Cells were treated with 1.0% Brij 98, and post-nuclear fraction was subjected to density gradient centrifugation as described in Methods. Equal volume from each fraction was subjected to immunoblot analysis to examine the distribution of APP, BACE1, and flotillin-1. IM, immature form; M, mature form. (**b**) Levels of APP, BACE1, PSEN1, and B4GALNT1 in DRM and DSM fractions. Equal amounts from fractions 2 and 3 (DRM) or 6–10 (DSM) were subjected to immunoblot analysis. (**c**) BACE1 contents in DRM. Relative BACE1 contents in DRM were quantified by setting M3-1 cells at 1 based on the result of panel b. BACE1 levels were normalized by flotillin 1 expression in DRM. Data represent means ± s.d. (n = 4). **P < 0.01 by one-way ANOVA with Tukey-Kramer post hoc test. (**d**) Relative BACE1 distribution. % BACE1 in DRM was quantified by the following equation: (BACE1 in DRM)/(BACE1 in DRM and DSM) × 100. Data represent means ± s.d. (n = 4). Statistical analysis was performed by one-way ANOVA with Dunnett post hoc test, and statistical significance was not detected.

Both the β-site and the γ-site cleavages of APP occur in lipid rafts^31^,^32^, membrane domains enriched in cholesterol and gangliosides^33^. Localisation of more BACE1 in such domains is expected to increase the generation of β-CTF. To test this possibility, we next sought to determine whether detergent-resistant membrane domains (DRM) contain more BACE1 in B4GALNT1-expressing cells. We lysed cells with the non-ionic detergent Brij 98, and subjected cell lysates to density gradient ultracentrifugation. The distribution of APP, BACE1 and flotillin-1 in cells expressing each ganglioside is shown in Fig. 7a. As expected, the DRM marker protein flotillin-1 was enriched in fractions 2 and 3 in all cell lines, while the majority of immature APP was recovered in fractions 6–10. Based on this result, we designated fractions 2 and 3 as DRM and fractions 6–10 as detergent-soluble membrane (DSM) fractions. BACE1 contents in DRM were markedly increased in GM1- and GM2-expressing cells compared to GM3-expressing cells (Fig. 7b,c) although BACE1 distribution pattern between DRM and DSM was not altered among the transfectants examined (Fig. 7d). A subtle, but non-significant increase in DRM-associated BACE1 was also observed in GD2-expressing cells. On the other hand, PSEN1 localisation to DRM was unchanged among the cells tested. These results suggest that higher expression of BACE1 in cells expressing B4GALNT1 leads to the increased localisation of this protein in lipid rafts.

## Discussion

A number of studies showed that gangliosides are involved in the pathophysiology of AD^11^,^12^. In addition to a well-known role of GM1 and other gangliosides in Aβ deposition^13^,^14^,^15^, gangliosides modulate APP processing^17^,^22^,^23^,^24^, but little is known about how gangliosides work. Our data suggest a hitherto undisclosed link between APP processing and ganglioside metabolism. We showed that the expression of B4GALNT1, an essential glycosyltransferase for synthesizing complex gangliosides abundantly expressed in the brain, increases BACE1 expression by inhibiting its lysosomal degradation, thereby promoting the β-site cleavage of APP. In contrast to forced expression of B4GALNT1, adding the gangliosides synthesized through this enzyme (including GM2, GM1, and GD2) exogenously did not produce such effects on BACE1 expression and APP processing. Our current results thus favor a model in which B4GALNT1 expression regulates BACE1 stability and activity, while the possibility that gangliosides containing a β1,4-linked GalNAc residue are involved in the events cannot be excluded.

BACE1 is the primary protease that cleaves APP at the β-site, which constitutes the first step in Aβ production. In addition to Aβ, β-CTF is found accumulated in the early stage of AD^34^. Moreover, BACE1 expression is increased in the brain of AD patients^35^,^36^,^37^. Blocking BACE1 is thus considered as a potential strategy to prevent AD onset and/or development^4^. BACE1 protein expression is regulated at transcriptional and post-transcriptional levels^27^,^38^. Our results showed that the expression of B4GALNT1 increases BACE1 protein without affecting its mRNA level. BACE1 protein degradation is regulated by intracellular transport to the lysosome where this protein is degraded^27^. Golgi-localized γ-ear-containing ARF binding (GGA) proteins 1–3 play an important role in BACE1 trafficking by interacting with the cytosolic domain of BACE1^39^. GGA1 and GGA3 have distinct roles in this transport pathway; GGA1 directs BACE1 protein to the trans-Golgi network (TGN) for escaping from degradation in the lysosome^40^, whereas GGA3 regulates endosome-to-lysosome transport of BACE1 for degradation^41^. Consequently, deficiency in GGA3 results in the stabilisation of BACE1, which promotes amyloidogenic processing of APP^41^. Importantly, GGA3 protein expression inversely correlates to BACE1 expression in the brain from AD patients^41^,^42^. In addition to GGA proteins, other proteins including reticulons^43^, sorting nexin-6^44^ and ARF6^45^ are also involved in the BACE1 trafficking. Furthermore, a recent work showed that the glycosyltransferase GnT-III, which catalyzes the addition of bisecting *N*-acetylglucosamine on BACE1, leads to the stabilisation of BACE1 by blocking its transport to the lysosome^46^. How B4GALNT1 expression modulates BACE1 stability is currently unknown, and thus we can only offer certain speculation. BACE1 is found in both the endosomes and the TGN. An intracellular compartment where BACE1 cleaves APP has been the subject of much investigation, yet the precise location remains unsettled. It has been reported that BACE1 acts both in the early endosome^45^ and the TGN^47^. B4GALNT1 resides and produces GM2 and GD2 in the TGN^48^ where substantial amounts of BACE1 localize. Since the addition of exogenous gangliosides to cell surface did not alter BACE1 stability, our data suggest that B4GALNT1 protein or newly synthesized gangliosides containing a GalNAc residue acts to modulate BACE1 stability in the TGN. Further studies are needed to directly determine whether B4GALNT1 plays a role in BACE1 stability. Transport of BACE1 from the endosome to the TGN sequesters this protein from the lysosomal degradation, leading to an increase in BACE1 protein and activity^41^. Therefore, B4GALNT1 or its products could increase BACE1 protein located in the TGN, which could directly promote the β-cleavage in this compartment or could serve as a pool for the endosomal BACE1.

Changes in brain ganglioside composition were repeatedly observed in AD patients^12^,^18^,^19^,^20^,^21^. The changes commonly found include an increase of GM2 and GM1^12^. GM1 is one of the major gangliosides in the brain, whereas GM2 is rarely present in this tissue. How these changes are produced in the brain is not fully understood. It was reported that skin fibroblasts isolated from AD patients display the enhanced catabolism of GM1 by the lysosomal enzyme acid β-galactosidase, leading to an increase in GM2 and GM3^49^. This observation may partly explain how AD brains contain more GM2. However, the increase of GM1 cannot be explained by the activation of this enzyme. It may be possible that the up-regulation of B4GALNT1 expression and reduction of ST8SIA1 expression increases GM2 and GM1. Although earlier works have shown the changes in ganglioside composition in AD, whether the expression of B4GALNT1 and other glycosyltransferases involved in ganglioside synthesis is altered in the brain from AD patients and/or model mice is essentially unknown at present. It is therefore important to clarify this issue in the future.

In the current work, we revealed that B4GALNT1 expression positively regulates BACE1 expression and the β-site cleavage of APP by blocking the lysosomal degradation of BACE1. Previous works demonstrated a critical role of BACE1 trafficking in the fate of this protein and in its activity. In the future, how the previously identified factors that regulate BACE1 trafficking modulate its degradation should be tested in the presence or absence of B4GALNT1 expression. It will be also interesting to evaluate roles of B4GALNT1 in APP processing in a cell type-specific manner *in vitro* and *in vivo*.

## Methods

### Reagents and antibodies

The γ secretase inhibitor DAPT was obtained from Peptide Institute, Inc. Cycloheximide, MG-132, chloroquine, Brij 98 were from Sigma-Aldrich. Glycosphingolipids (LacCer, GM3, GM2, GM1, GD2, and GD1b) were from Sigma-Aldrich and/or Wako. GD3 and bovine brain gangliosides were from Calbiochem. Human/Rat β Amyloid (40) (#294–62501) and Human/Rat β Amyloid (42) (#290–62601) ELISA Kits were purchased from Wako. Cholera toxin B subunit (CTxB) biotin conjugate was from List Biological Laboratories. The following antibodies were obtained from commercial sources as follows: anti-APP mouse monoclonal antibody (mAb) (clone 22C11, MAB348) and anti-presenilin-1 mouse mAb (clone PS1-loop, MAB5232) from Millipore; anti-APP rabbit mAb (clone Y188, #1565-1) from Epitomics; anti-human amyloid β (N) mouse mAb (clone 82E1, #10323) from Immuno-Biological Laboratories, anti-BACE1 rabbit mAb (clone D10E5, #5606) and HRP-conjugated anti-mouse or anti-rabbit IgG antibody from Cell Signaling Technology; anti-flotillin-1 rabbit polyclonal antibody (H-104, sc-25506), anti-B4GALNT1 mouse mAb (clone C5, sc-376505) and anti-c-Myc mouse mAb (clone 9E10) from Santa Cruz Biotechnology; anti-GM3 mouse mAb M2590 from Nippon BioTest Laboratories. Anti-GD3 mAb R24 and anti-GM2 mAb 10–11 were kindly provided from Dr. Lloyd J. Old (Memorial Sloan-Kettering Cancer Center) and from Dr. P. O. Livingston, respectively. Anti-GD1a mAb D226, anti-GD2 mAb 220-51, anti-GD1b mAb 370, and anti-GT1b mAb 549 were previously generated in our laboratory^50^,^51^,^52^. FITC-conjugated anti-mouse IgG and FITC-conjugated anti-mouse IgM were from Cappel and Zymed, respectively. PE-conjugated anti-mouse IgM was from Sigma-Aldrich.

### Cell lines and cell culture

SK-MEL-28-N1 (referred as to N1 hereafter), a GD3-negative cell line isolated from SK-MEL-28 human melanoma cells, was established previously^53^, and was used as recipient cells for the glycan remodelling. The major ganglioside in N1 cells is GM3. N1 cells stably expressing GD3 (G5 and G11) and mock transfectants (V4 and V9) were generated previously in our lab^25^. GM2-expressing and GD2-expressing cell lines were established by transfecting N1 cells and GD3-expressing cells with human B4GALNT1 cDNA expression vectors^54^, respectively. DNA transfection was conducted by using Lipofectamine 2000 (Invitrogen) according to manufacturer’s protocol. Afterwards, GM2- and GD2-expressing cells were sorted by flow cytometry using anti-GM2 mAb and anti-GD2 mAb, respectively, and clones were then obtained by limiting dilution. GM1-expressing cell lines were established by trasfecting GM2-expressing cells with B3GALT4 cDNA^51^ as above, followed by flow cytometric isolation of cells that bind biotin-CTxB. These melanoma cell lines were maintained in Dulbecco’s modified Eagle’s medium (DMEM) containing 7.5% FBS, 1% penicillin and streptomycin mixture (Gibco) and 400 μg/ml of G418 at 37 °C in an incubator with 5% CO_2_. For experiments, cells were seeded into 6-well plates at a density of 2 × 10^5^ cells per well or 100-mm dishes at a density of 1 × 10^6^ cells per dish.

### Loading cells with gangliosides

Gangliosides were dissolved in chloroform and methanol (1:1). Appropriate amounts of ganglioside were transferred to a fresh glass tube and dried under nitrogen gas. Ganglioside was dissolved in warm serum-free DMEM at a final concentration of 30 μM. Cells were then incubated in serum-free medium containing each ganglioside for 21 h at 37 °C. The ganglioside concentration and time of treatment used in this study was determined according to previous reports^17^,^22^,^24^,^55^. The incorporation and cell surface expression of gangliosides added was confirmed by flow cytometry and by thin layer chromatography (TLC) as described below.

### FACS analysis

Cell surface expression of gangliosides was analysed by flow cytometry. The following antibodies/agents were used to detect cell surface gangliosides; GM3, M2590; GM2, 10–11; GM1, biotin-conjugated CTxB, GD3, R24; GD2, 220–51; GD1b, 370; GT1b, 549. Cells detached by trypsin and EDTA were incubated with a primary antibody for 1 h on ice. After washing three times with ice-cold PBS, cells were then incubated with FITC-conjugated secondary antibody (anti-mouse IgG, anti-mouse IgM or avidin) or PE-conjugated anti-mouse IgM (for M2590 only) for 1 h on ice. Cells were subjected to flow cytometry using FACSCalibur (BD Biosciences). Mean fluorescence intensity was analysed with CellQuest software (BD Biosciences).

### Analysis of ganglioside by TLC

Total cell lipids were extracted by chloroform/methanol (2:1 and 1:1, v/v). Acidic fraction (containing gangliosides) was prepared as described^26^. Acidic fraction or total lipids were spotted onto an HPTLC plate (Merck) and separated by a solvent system of chloroform/methanol/0.2% CaCl_2_ (55:45:10). A mixture of bovine brain ganglioside and GM3 (and GM2) was used as a standard. Gangliosides were detected by resorcinol spray.

### Quantitative RT-PCR (qRT-PCR)

Total RNA was isolated using TRIzol Reagent (Invitrogen) according to manufacturer’s instruction. cDNA was synthesized using Reverse Transcriptase Kit with oligo(dT) primer (Invitrogen). Quantitative PCR was performed using SsoAdvanced SYBR Green Supermix (Bio-Rad). Primers for APP, BACE1 and PSEN1 were shown in Supplemental Table S1. The mRNA expression levels were calculated by ∆∆CT method using GAPDH or β-actin mRNA as an internal standard.

### Immunoblot

Cells seeded in 6-well plates were grown for 1–2 days at 37 °C. Cells were lysed with RIPA buffer (50 mM Tris-HCl pH 8.0, 150 mM NaCl, 2 mM EDTA, 1% NP40, 0.1% SDS, 0.5% sodium deoxycholate, and 0.2% protease inhibitor cocktail (Sigma-Aldrich)), and the cell lysates were then spun at 16,000 g for 3 min at 4 °C. Resultant supernatant was used as whole cell lysate. Protein contents were determined by BCA assay (Thermo). Equal amounts of protein were subjected to SDS-PAGE followed by immunoblot analysis according to a standard procedure. To detect CTFs of APP, cell lysate was subjected to 12%-Tricine SDS-PAGE as described^56^. Protein expression was quantified by ImageJ software.

### Cell fractionation

DRM was isolated as described previously^57^,^58^ with modifications. Cells grown in two 100-mm dishes were treated with 1% Brij 98 (600 μl per dish) for 10 min at 37 °C in TNE buffer (25 mM Tris-HCl, pH 7.6, 150 mM NaCl and 5 mM EDTA) containing 0.2% protease inhibitor cocktail (Sigma-Aldrich). Cell lysate was homogenized by five passages through a 26-gauge needle, and cell homogenate was spun twice at 1,000 g for 5 min at 4 °C to obtain post-nuclear supernatant. One ml of post-nuclear supernatant was mixed with 1 ml of 80% sucrose to adjust the sucrose concentration to 40%, and placed at the bottom of an ultracentrifuge tube (Beckman Coulter). 30% sucrose (1.25 ml) was layered, and 5% sucrose (0.75 ml) was then layered on the top. Samples were centrifuged at 104,000 g for 16 h at 4 °C in a MLS-50 rotor (Beckman). Ten 0.4 ml fractions were collected from the top.

### Statistical analysis

Data were presented as mean ± s.d. Statistical analysis was performed using the two-tailed, unpaired Student’s *t* test, or using a one-way ANOVA with Tukey-Kramer or Dunnett post hoc test as specified in the individual figure legends. *P* values smaller than 0.05 were considered statistically significant.

## Additional Information

**How to cite this article**: Yamaguchi, T. *et al*. Expression of B4GALNT1, an essential glycosyltransferase for the synthesis of complex gangliosides, suppresses BACE1 degradation and modulates APP processing. *Sci. Rep.* **6**, 34505; doi: 10.1038/srep34505 (2016).

## Supplementary Material

Supplementary Information
