# Supplementary material for: Expression of B4GALNT1, an essential glycosyltransferase for the synthesis of complex gangliosides, suppresses BACE1 degradation and modulates APP processing
Source: Sci Rep. 2016 Sep 30;6:34505. doi: 10.1038/srep34505 (PMC5043288; doi:10.1038/srep34505)
Supplement: Supplementary Information [file srep34505-s1.pdf]

Supplemental Information

Expression of B4GALNT1, an essential glycosyltransferase for the synthesis of complex gangliosides, suppresses BACE1 degradation and modulates APP processing

Tokiaki Yamaguchi[1], Yoshio Yamauchi[1,*], Keiko Furukawa[2], Yuhsuke Ohmi[1], Yuki Ohkawa[1,2], Qing Zhang[1], Tetsuya Okajima and Koichi Furukawa[1,2,*]

[1]Department of Biochemistry II, Nagoya University Graduate School of Medicine, 65 Tsurumai, Showa-ku, Nagoya 466-8550, Japan
[2]Department of Biomedical Sciences, Chubu University College of Life and Health Science, Kasugai 487-8501, Japan

*Corresponding authors
Yoshio Yamauchi, Department of Biochemistry II, Nagoya University Graduate School of Medicine, 65 Tsurumai, Showa-ku, Nagoya 466-8550, Japan, Tel: +81-52-744-2068, E-mail: yyoshio@med.nagoya-u.ac.jp
Koichi Furukawa, Department of Biomedical Sciences, Chubu University College of Life and Health Sciences, 1200 Matsumoto, Kasugai 487-8501, Japan, Tel: +81-568-51-9512, E-mail: koichi@isc.chubu.ac.jp

Supplemental Table 1

Primers used for qRT-PCR

Gene	primer	Sequence
APP	Forward	5'-TGCCGACCGAGGACTGACCAC-3'
	Reverse	5'-CGGCGTCAACCTCCACCACAC-3'
BACE1	Forward	5'-CCCGCAGACGCTCAACATCC-3'
	Reverse	5'-TGGCAGCAATGTTGGCACGC-3'
PSEN1	Forward	5'-GACAATAGAGAACGGCAGGAGCACA-3'
	Reverse	5'-TGGTAGCCACGACCACCACCA-3'

Supplemental Figure 1

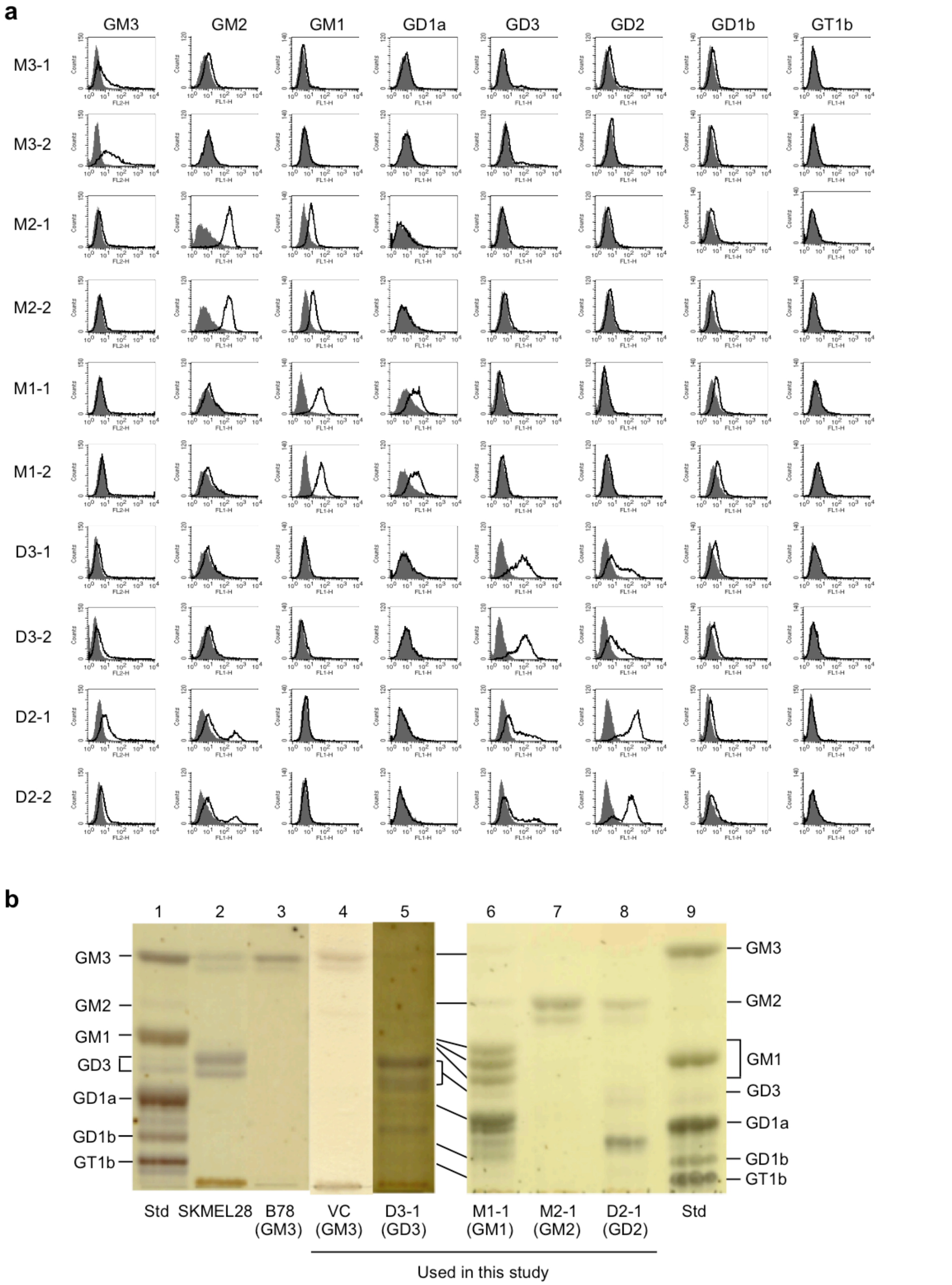

Supplemental Figure 1. Expression of gangliosides in melanoma cell lines used in this study.

(**a**) Cell surface expression of the indicated gangliosides in each cell line was examined as described in Methods.

(**b**) The composition of gangliosides. Cellular lipids were extracted from the indicated cells and acidic fraction was obtained. Gangliosides were analyzed by TLC as described in Methods. A part of the results (lanes 1 – 3) was reported previously (ref. 26).

Supplemental Figure 2

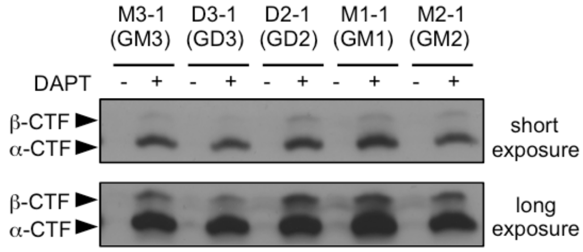

Supplemental Figure 2. Effect of DAPT on α- and β-CTFs.

Cells expressing GM3 (M3-1), GD3 (D3-1), GD2 (D2-1), GM1 (M1-1) or GM2 (M2-1) were incubated in the presence or absence of 1 μM DAPT for 12 h. Expression of α- and β-CTFs were probed with anti-APP mAb Y188.

Supplemental Figure 3

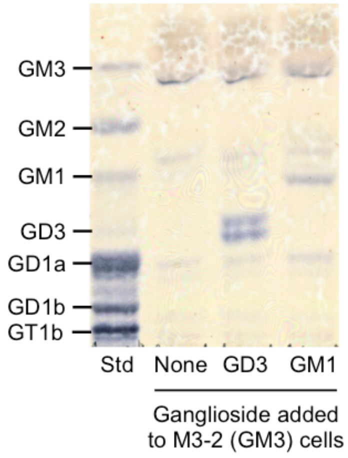

Supplemental Figure 3. The incorporation of GD3 and GM1 added exogenously into GM3-expressing cells.

Cells expressing GM3 (M3-2) were incubated without or with 30 μM GD3 or GM1 for 21 h. Afterward, total cell lipids were extracted and separated by TLC as described in Methods. Gangliosides were detected by resorcinol.
